# Recent Advances in Understandings Towards Pathogenesis and Treatment for Intrauterine Adhesion and Disruptive Insights from Single-Cell Analysis

**DOI:** 10.1007/s43032-020-00343-y

**Published:** 2020-10-30

**Authors:** Ross Ka-Kit Leung, Yixin Lin, Yanhui Liu

**Affiliations:** 1 Dongguan Maternal and Child Health Care Hospital, Dongguan, 523120 China; 2 Dongguan Institute of Reproductive and Genetic Research, Dongguan, 523120 China

**Keywords:** Intrauterine adhesion (IUA), Cell heterogeneity, Cell-cell interaction, TGF-β1/SMAD pathway, Molecular mechanism

## Abstract

Intrauterine adhesion is a major cause of menstrual irregularities, infertility, and recurrent pregnancy losses and the progress towards its amelioration and therapy is slow and unsatisfactory. We aim to summarize and evaluate the current treatment progress and research methods for intrauterine adhesion. We conducted literature review in January 2020 by searching articles at PubMed on prevention and treatment, pathogenesis, the repair of other tissues/organs, cell plasticity, and the stem cell–related therapies for intrauterine adhesion. A total of 110 articles were selected for review. Uterine cell heterogeneity, expression profile, and cell-cell interaction were investigated based on scRNA-seq of uterus provided by Human Cell Landscape (HCL) project. Previous knowledge on intrauterine adhesion (IUA) pathogenesis was mostly derived from correlation studies by differentially expressed genes between endometrial tissue of intrauterine adhesion patients/animal models and normal endometrial tissue. Although the TGF-β1/SMAD pathway was suggested as the key driver for IUA pathogenesis, uterine cell heterogeneity and distinct expression profile among different cell types highlighted the importance of single-cell investigations. Cell-cell interaction in the uterus revealed the central hub of endothelial cells interacting with other cells, with endothelial cells in endothelial to mesenchymal transition and fibroblasts as the strongest interaction partners. The potential of stem cell–related therapies appeared promising, yet suffers from largely animal studies and nonstandard study design. The need to dissect the roles of endometrial cells, endothelial cells, and fibroblasts and their interaction is evident in order to elucidate the molecular and cellular mechanisms in both intrauterine adhesion pathogenesis and treatment.

## Statement of Significance

**Table:** 

Problem or issue	What is already known	What this paper adds
IUA causes severe gynecological disorders, but the prevention strategies and treatments have been unsatisfactory and improvements are limited. Current knowledge on IUA pathogenesis was mostly derived from tissue studies without considering the multicellular structures and their orchestration in the endometrial tissue and in-depth mechanistic investigations. Development of stem cell–related therapies is limited.	TGF-β1/SMAD pathway is most likely to play a central role in IUA pathogenesis while other candidates were listed. Molecules and signaling pathways associated with cell plasticity were also listed. Stem cell–related therapies were proved to be effective in animal and clinical studies.	Analyses of uterine cell heterogeneity and cellular expression profile indicate that the previous research methods on IUA pathogenesis may miss important details. Analysis of cell-cell interaction suggested that injured endometrial cells may communicate with other cells via endothelial system and fibroblasts could be the first few to respond and finally lead to fibrosis. The repairing effects of stem cell–derived vesicles are worth exploration and the list of candidates are summarized in this review.

## Introduction

Intrauterine adhesion (IUA) is a gynecological disease characterized by partial or full adhesion of the anterior and posterior walls of the uterine cavity after endometrial injury. The clinical manifestations include menstrual irregularities, amenorrhea, infertility, placenta previa, recurrent miscarriage, premature delivery, placental adhesions, difficulties in embryo implantation, and abnormal placental development, but there are also asymptomatic intrauterine adhesions [1]. The main causes include miscarriage curettage [2], postpartum curettage [2], myomectomy [3], and endometrial ablation [4]. The German doctor Fritsch reported the first case of IUA in 1894, and the Israeli obstetrician and gynecologist Asherman made a complete description of the disease for the first time in 1948 [5]. Therefore, IUA is also known as Asherman’s syndrome (AS).

The treatment for IUA is mainly hysteroscopic surgery followed by re-adhesion prevention. However, Chen et al. [6] found that the re-adhesion rate after treatment of mild and moderate IUA was 30% and as high as 62.5% for severe cases. Moreover, the pregnancy rate was only 22.5~33.3% [6], which is far from satisfactory.

Besides IUA, there are two related pathologies worth paying attention to: endometriosis and absolute uterine factor infertility (AUFI). Endometriosis is on one extreme a condition that endometrial cells grow outside the uterine cavity as functional glands and stroma. They undergo cyclic proliferation and breakdown, similar to what happens in the normal endometrium. The difference is that the internal bleeding usually cannot be cleared, which leads to an outcome as identified in IUA, and inflammations result in scar formation and adhesions during repair. A comprehensive review about the molecular and cellular pathogenesis of endometriosis can be found in [7]. The other extreme is a condition without the uterus at all or a nonfunctional one. Attempts were made to resume the functionalities by uterus transplantation and several livebirths have already been reported [8–10]. In particular, uterine tissue transplants were employed to assess both endometriosis and uterine repair of a partially defect uterus [11, 12]. Key insights from these studies into IUA research include the following: (1) endometrial functionalities can be reconstructed, which is reflected by the pathological conditions of endometriosis, and uterine tissue transplant where pregnancy was observed in the once nonfunctional uterus; and (2) the usefulness of cellular models that elucidate the fates of cell differentiation and functional response towards pathological and pregnant conditions. We conducted literature review in January 2020 by searching articles at PubMed on prevention and treatment, pathogenesis, the repair of other tissues/organs, cell plasticity, and the stem cell–related therapies for IUA. A total of 110 articles were selected for review.

## Prevention and Treatment Options for IUA and Their Effectiveness

Meta-analysis studies did not suggest the usefulness of interventions for IUA. Bosteels et al. [13] reported that no bio-gel methods could conclusively increase pregnancy rate after undergoing hysteroscopic surgery. Healy et al. [14] showed that no strong evidence to prove that hyaluronic acid gel, polyethene oxide-carboxymethyl cellulose sodium gel, or estrogen could prevent IUA. Johary et al. [15] suggested that estrogen be combined with other ancillary treatment methods, for example, intrauterine devices (IUDs), Foley catheter, hyaluronic acid gel, or amnion graft, to reach better pregnancy rates and live birth rates. Likewise, IUD needs to be combined with other ancillary treatments such as hormone therapy, Foley catheter, hyaluronic acid gel, or amnion graft to obtain maximal outcomes [16]. Khan and Goldberg [17] suggested that stem cell therapy is a significantly better choice than current strategies. Kou et al. [18] suggested that using IUDs/gels to deliver therapeutic factors (such as hormones and/or stem cells) to the injured uterine site may be an effective prevention method, which lacked further verification.

Studies analyzed by Johary et al. [15] found that combining estrogen and other adjunctive therapies have found that the pregnancy rate after treatment is between 8 [19] and 90% [20], and the live birth rate is between 5.2 [17] to 70% [20]. This treatment outcome was better than using estrogen alone [21–23]. Similarly, IUD combined with other adjunctive treatments had a better outcome than using IUD only, but the conception rate and live birth rate were also unstable [16]. Therefore, there is a pressing need for the development of effective and stable prevention and treatment methods for IUA. Due to the limited understanding of IUA pathogenesis and the normal repair mechanism of damaged endometrium, it is hard to develop targeted methods to efficiently promote the regeneration of endometrium. The study on IUA pathogenesis should be the basis of the development of new prevention and treatment methods for IUA.

## IUA Pathogenesis

### Research Progress and Its Limitations

**Fig. 1:**
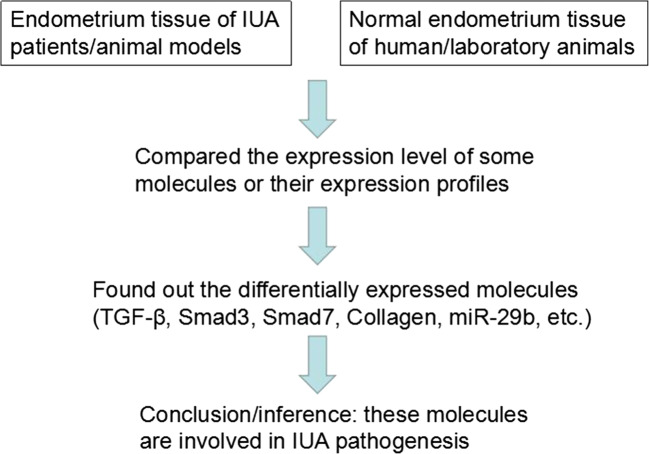
Mainstream methodology for the current research in IUA pathogenesis

**Fig. 2:**
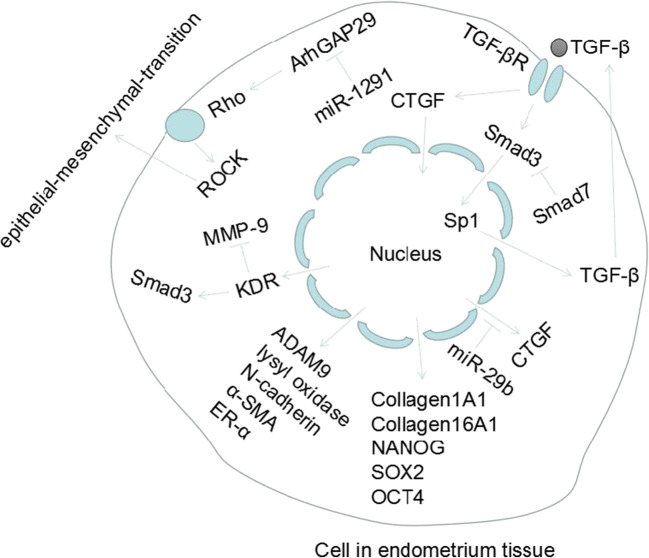
Potential molecular mediators involved in IUA pathogenesis

IUA is a phenomenon that fibrosis occurs in the damaged endometrium without adequate self-repair [22, 24, 25]. Several studies reported highly expressed fibrotic markers such as TGF-β in the endometrial tissue of IUA patients or animal models [26–31]. The TGF-β1/SMAD pathway was shown to play a leading role in the molecular network that induces fibrosis in various organs [32]. Current research on IUA pathogenesis has mainly been based on this pathway. Reviewing their methodologies (Fig. 1), previous studies mainly identified differentially expressed proteins/miRNAs/mRNAs between the endometrial tissue of the IUA patients and normal individuals, or IUA and normal experimental animal models, and inferred that these molecules are involved in IUA pathogenesis (Fig. 2).

Salma et al. [31] showed that the levels of TGF-β1 and SMAD3 in IUA patients or experimental animal models had been higher than those in the control group, while SMAD7 was significantly reduced. Therefore, they speculated that TGF-β1/SMAD3/SMAD7 signaling pathway is the main regulator of IUA pathogenesis. Xue et al. [27] compared the endometrial tissue of IUA patients with that of normal individuals and found that the expression of TGF-β and CCN2 in the former was higher and suggested that TGF-β and CCN2 were related to IUA pathogenesis. They also found that the activity of the NF-κB pathway is positively correlated with TGF-β and CCN2 expression. When the NF-κB signaling pathway was inhibited with SN50, lower expression of TGF-β resulted. Hence, the conjectured activation of the NF-κB pathway is also related to the formation of IUA. Wang et al. [33] also reported that NF-κB was highly expressed in endometrial tissues of IUA patients and therefore a putative pathogenic factor for IUA. Chen et al. [30] found that the expression of KDR in IUA tissues was significantly higher than that of the normal counterpart, and also the expression of KDR was positively correlated with the severity of IUA. Silencing KDR could upregulate MMP-9 and affect TGF-β1/SMAD pathway to inhibit the occurrence and development of IUA, so KDR was thought to play an important role in the formation of IUA. Xiao et al. [34] found that in LPS-induced IUA rat models, against the control group, the expression of SOX2, NANOG, and OCT4 increased significantly. They also found that in IUA patients, the expression of NANOG was significantly higher than that of normal people. Collectively, SOX2, NANOG, and OCT4 might be involved in the pathogenesis of IUA. Guo et al. [29] found that the TGF-β1/BMP7/SMAD pathway and epithelial-mesenchymal transition (EMT) could promote the development of IUA. EMT has also been found associated with development of other pathological conditions including Zhou et al. [26] who found that the expressions of TGF-β1, MMP-9, and ERα in endometrial tissues of IUA patients or rat models were significantly higher than those of the control group. Moreover, the expression of these molecules was significantly higher in patients with severe than mild or moderate IUA. They proposed that abnormal activation of fibrosis and overexpression of ERα may be involved in the formation of mild and moderate IUA, and the SDF1/CXCR-4 axis may also be involved in the immune response during IUA formation. Since there were few studies on the immune response in IUA pathogenesis, the role of the SDF-1/CXCR-4 axis in IUAs as an inflammatory mediator requires further verification.

The regulatory effects of miRNAs on the formation or development of IUA were studied. Li et al. [35] found that the expression of COL1A1, α-SMA, CTGF, and the transcription factor SP1 increased in the endometrial tissue of the rat IUA model, while the expression of E-cadherin and microRNA-29 (miR-29) decreased. The study further found that miR-29 inhibited tissue fibrosis by downregulating the Sp1-TGF-β1/SMAD-CTGF pathway. Liu et al. [36] applied microarray analysis to profile the mRNA and miRNA expression of endometrial tissues. There were 26 miRNAs and 1180 mRNAs significantly differentially expressed between the patients with severe IUA and normal individuals. Real-time PCR experiments confirmed that miR-513a-5p and miR-135a-3p were upregulated, while miR-543 was downregulated. Their target genes CDH2 (N-cadherin) and COL16A1 (collagen 16A1) were upregulated, while ADAM9 and lysyl oxidase were downregulated. How these molecules participate in the formation or development of IUA were unanswered though. Xu et al. [37] found that miR-1291, as an upstream molecule of ArhGAP29, promotes fibrosis of the endometrium by negatively regulating the RhoA/ROCK1 EMT pathway.

In addition to the abovementioned studies on endometrial tissues of IUA patient or animal models, there were also studies using human endometrial stromal cells as cell models to study the IUA pathogenesis. Li et al. [38] validated their findings of miRNA-29b inhibition of the development of IUA by regulating the TGF-β1/SMAD pathway in human endometrial stromal cells. Ning et al. [25] used microarray to evaluate the miRNA expression profiles of endometrial tissue and normal endometrial tissue in patients with IUA and validated that miRNA-326 could inhibit endometrial fibrosis by inhibiting the TGF-β1/smad3 pathway. Therefore, miRNA-29b and miRNA-326 were potential candidates for IUA treatment and further studies. The recent studies on IUA pathogenesis are summarized in the Table 1.

**Table 1:** Summary of previous work on IUA pathogenesis

Species	Tissue	Sample size		Cell line/cell model	Molecules/pathways associated with IUA pathogenesis	References
		Human	Animal model			
Human; rabbit	Blood/uterine tissue	60 patients and 30 fertile women	18 IUA rabbit models and 18 mature female fertile rabbits	–	Smad3; Smad7; TGF-β1	[31]
Human; rat	Endometrial tissue	40 intrauterine adhesion tissues and 20 normal endometrium tissues	30 IUA rats (phenol mucilage treatment), 15 rats for sham group (with mock treatment), and 15 rats for normal group (with no treatment)	–	NF-κB	[33]
Human; rat	Endometrial tissue	92 patients and 86 women in control group	50 rats were divided into control group, sham group, model group, NC-siRNA group, and KDR-siRNA group, with 10 rats in each group	–	KDR; TGF-β1/SMAD pathway; MMP-9	[30]
Human; mouse	Women endometrial tissue; mouse uterine horns	19 women with IUA and 16 disease-free women as control group	Not indicated	–	SOX2; NANOG; OCT4	[34]
Rat	Endometrial tissue	–	6 rats in IUA group and 6 in sham group	–	TGF-β1/BMP7/SMAD pathway; epithelial-mesenchymal transition (EMT)	[29]
Human; rat	Endometrial tissue	76 IUA patients and 20 samples of normal endometrium	70 rats in experimental group and 10 in control group	–	TGF-β1; MMP-9; ERα; SDF-1/CXCR-4 axis	[26]
Rat	Endometrial tissue	12 rat IUA models, 4 rats in sham-operated group, and 4 in control group	–	–	miR-29b; Sp1/TGF-β1/SMAD-CTGF	[35]
Human	Endometrial tissue	3 patients with severe IUA (the sample size of normal endometrium was not indicated)	–	–	miR-513a-5p; miR-135a-3p; miR-543; N-cadherin; collagen 16A1; ADAM9; lysyl oxidase	[36]
Human; mouse	Endometrial tissue	39 patients with IUAs and 28 normal control cases	12 mice were divided into three groups: IUAs (*n* = 3), miR-1291 antagomir (*n* = 3), and NC (*n* = 3)	–	miR-1291; ArhGAP29; RhoA/ROCK1 EMT pathway	[37]
Human	Endometrial tissue	Not indicated	–	Primary endometrial stromal cells (ESCs)	miR-29b; TGF-β1/SMAD pathway	[38]
Human	Endometrial tissue	70 endometrium tissue from IUA patients, 15 from patients with uterine septum, and 15 normal endometrium	–	RL95–2	TGF-β; CCN2; NF-κB pathway	[27]
Human	Endometrial tissue	30 endometrial tissues from IUA patients and 15 normal endometrial tissues	–	Primary endometrial stromal cells (ESCs)	miRNA-326; TGF-β1/Smad3	[25]

Zhu et al. [39] suggested that the Hippo pathway may form a complex molecular network with the TGF-β and Wnt pathways to control the development of endometrial fibrosis and their belief was based on (1) the important role of TGF-β pathway in IUA pathogenesis, (2) the role of Hippo pathway in the development of normal endometrium [40, 41], (3) the important role of Hippo in fibrosis of other tissues [42, 43], and (4) the crosstalk of Hippo pathway with the TGF-β and Wnt pathways in other tissue or cell models [42, 44–48]. However, no direct evidence indicating the role of Hippo pathway in IUA pathogenesis was presented. Therefore, whether the Hippo pathway is the response or driver needs further verification.

In spite of a large number of gene expression and pathway analysis studies on IUA, the multicellular structures in the endometrium could undermine the validity of the aforementioned research findings. For example, single-cell RNA sequencing has unveiled immune system heterogeneity by identifying novel distinct immune cell subsets [49], which cannot be accomplished by tissue studies. Tumor heterogeneity such as the full spectrum of mutations can only be accurately studied by single-cell methods [50]. Note that the uterus consists of the uterine body and the cervix and intrauterine adhesion refers to the adherence of the endometrial surfaces with fibrotic scar. The endometrium is cell-rich, consisting of secretory, cilia, and stromal cells. Studies that take samples from the uterus do not only retrieve these cells, but also epithelial, myometrium, endothelial, and possibly also some other cells. For example, epithelial and stromal cells represent two distinct groups and their molecular markers and response are different [51–53]. Single-cell methods are options to help pinpoint the underlying causes of IUA pathogenesis.

### Uterine Cell Heterogeneity

Ignoring the complex tissue structure of the uterus and the distinct roles that different cell types may play in the pathogenesis of IUA can invalidate the comparison between the normal uterine and IUA tissues to study the pathology and mechanisms. Single-cell sequencing is a powerful technique to overcome the limitations. Han et al. [54] performed single-cell sequencing on each organ/tissue sample of the Chinese Han population, which provided valuable data for revealing the complex cell types in each organ/tissue.

**Fig. 3:**
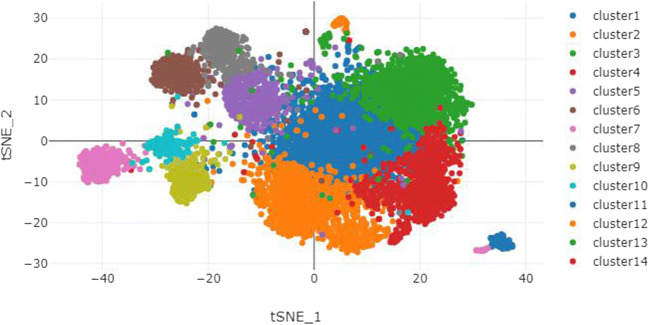
Various cell types in uterus. cluster1: endothelial cell_COL15A1 high; cluster2: endothelial cell_ESM1 high; cluster3: endothelial cell_IL6 high; cluster4: endothelial cell_SOCS3 high; cluster5: smooth muscle cell_MYL9 high; cluster6: stromal cell_RGS5 high; cluster7: fibroblast; cluster8: smooth muscle cell_PDK4 high; cluster9: smooth muscle cell_ACTG2 high; cluster10: stromal cell_ERRFI1 high; cluster11: endometrial cell; cluster12: M1 Macrophage; cluster13: T cell; cluster14: endothelial cell in EMT; cluster15: endothelial cell_FABP4 high; cluster16: Mast cell; cluster17: luminal epithelium

**Fig. 4:**
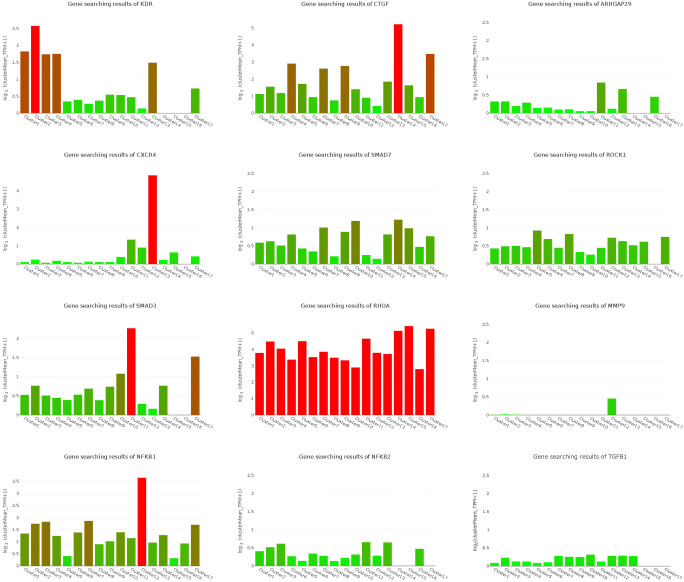
Uterine cellular expression profile related to IUA pathogenesis. cluster1: endothelial cell_COL15A1 high; cluster2: endothelial cell_ESM1 high; cluster3: endothelial cell_IL6 high; cluster4: endothelial cell_SOCS3 high; cluster5: smooth muscle cell_MYL9 high; cluster6: stromal cell_RGS5 high; cluster7: fibroblast; cluster8: smooth muscle cell_PDK4 high; cluster9: smooth muscle cell_ACTG2 high; cluster10: stromal cell_ERRFI1 high; cluster11: endometrial cell; cluster12: M1 macrophage; cluster13: T cell; cluster14: endothelial cell in EMT; cluster15: endothelial cell_FABP4 high; cluster16: mast cell; cluster17: luminal epithelium

We performed t-SNE analysis based on single-cell sequencing data of the uterus to distinguish multiple types (clusters) of cells in the uterus (Fig. 3), including six types of endothelial cells with different molecular markers, endometrial cells, fibroblasts, luminal epithelium, M1 macrophages, mast cells, three types of smooth muscle cells with different molecular markers, two types of stromal cells with different molecular markers, and T cells. For examples, in the six types of endothelial cells, endothelial cell_COL15A1 high denotes the endothelial cells expressing COL15A1 significantly compared with the other five, so that COL15A1 can be regarded as a marker of this type of endothelial cells. Similar concepts apply to smooth muscle and stromal cells. Cell heterogeneity is evident in the uterus at molecular level and this lays a solid foundation for studying the role of different cell types during the formation of IUA. In Fig. 4, the expression profiles of these genes varied a lot in different types of cells, which suggests the distinct roles of these cells during IUA formation. Taking the whole uterus as a whole for research could miss important information; for example, SMAD3 was high in endometrial cell while it was low in endothelial cell_FABP4 high, hinting us SMAD3 might not be associated with angiogenesis. Moreover, NFKB1 was high in M1 macrophage while it was low in endothelial cell_FABP4 high, resulting in “no changes” as a whole.

### Insufficient Cell Plasticity Model for IUA Pathogenesis

IUA is the consequence of endometrial fibrosis, so understandings towards the molecular mechanisms of fibrosis in other tissues are informative. We hypothesize that in the injured endometrium, insufficient functional cell renewal capacity will lead to exposure of the wound to pathogens and dead cells, infiltrating immune cells and other effector cells, and ultimately steer the emergent response of a large amount of extracellular matrix secreted to seal the wound, thus forming IUA. Similar phenomena have been observed in cardiac, hepatic, renal, and pulmonary fibrosis [32].

**Fig. 5:**
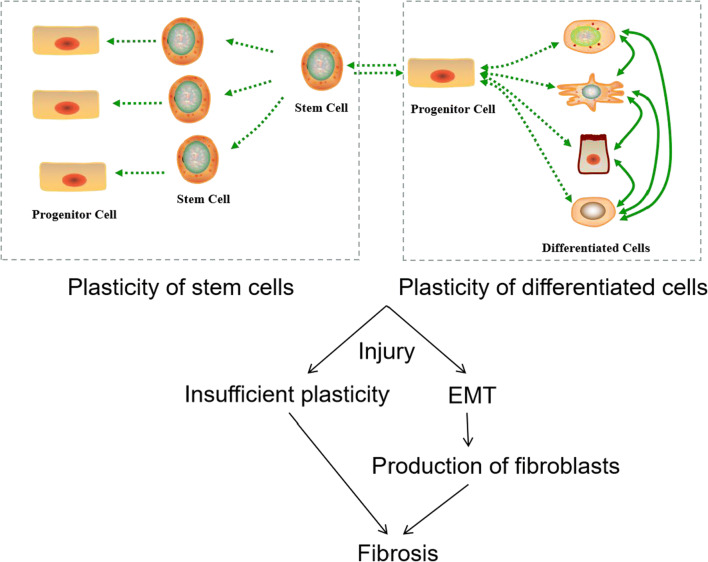
Schematic diagram of cell plasticity and differentiation

To test the above hypothesis, the mechanisms of self-repair after endometrial injury followed by the cause of insufficient self-repair ability should be studied. The molecular mechanisms leading to fibrosis in the endometrium should be one of the first few tasks to work on. While there is little research on exploring the mechanisms of self-repair after endometrial injury, studies on other epithelial tissues can shed some light [71]. In general, when tissue is traumatized without significant infection or inflammation occurs, the ability of self-healing mainly depends on the dedifferentiation/transdifferentiation ability of differentiated cells [72–80]. The molecules and signaling pathways associated with dedifferentiation and transdifferentiation are shown in Table 2. The insufficient cell plasticity model for IUA pathogenesis is as follows. Injured endometrial cells have limited self-repair capacity and are exposed to potentially hostile environments. When stimuli (such as immune response) induced by factors such as pathogens and dead cells reach a certain level, fibroblasts and/or other effector cells are activated to form fibrous tissue to seal the wound. We hypothesize that fibrous tissues between the wounds get connected when they are formed, and eventually cause adhesions between the walls of the uterine cavity (Fig. 5).

**Table 2:** Molecules and signaling pathways associated with differentiation and transdifferentiation

Pathway	Activation/inhibition	Starting cell and ending cells	Remarks	References
Notch	Inhibition	Mouse fibroblast → cardiomyocytes	–	[55]
JAK-STAT	Activation	Mouse neural stem cells/fibroblasts → iPSCs	–	[56]
	Inhibition	Mouse embryonic fibroblasts → cardiomyocytes	With expression of OSKM and Bmp4	[57]
TGF-β	Inhibition	Mouse embryonic fibroblasts → iPSCs	–	[58]
		Mesenchymal-type human fibroblasts → iPSCs	–	[58]
BMP	Activation	Mouse fibroblasts → cardiovascular progenitor cells (CPC)	With activation of Wnt and TGF-β pathway	[59–62]
Wnt	Activation	Mouse fibroblasts/neural stem cells → iPSCs	–	[63, 64]
	Inhibition	Mouse fibroblasts → cardiomyocyte	With SB431542	[60]
	Activated by CHIR99021	Mouse fibroblasts → cardiomyocyte	With Repsox (inhibiting TGF-β signaling), forskolin (increasing cAMP), and phosphodiesterase (PDE) 4 inhibitors (rolipram and cilomilast)	[65]
		Human fibroblasts → neurons	With inhibition of TGF-β signaling by SB431542 and transduction with Ascl1 and Ngn2	[66]
MAPK/ERK	Inhibited by PD0325901	Mouse neural progenitor cells → iPSCs	–	[67]
ROCK	Inhibited by Y-27632	Mouse fibroblasts → cardiomyocytes	–	[68]
		Human dermal fibroblasts → induced multipotent mesenchymal stem cell–like cells (iMSCs)	With SP600125 (JNK inhibitor), SB202190 (p38 inhibitor), Go 6983 (PKC inhibitor), PD0325901 (ERK1/2 inhibitor), and CHIR99021, with or without growth factors (TGF-β, bFGF, and LIF)	[69]
mTOR	Inhibited by Sox2	Mouse embryonic fibroblasts → iPSC	–	[70]

### Cell-Cell Interaction in Uterus

**Fig. 6:**
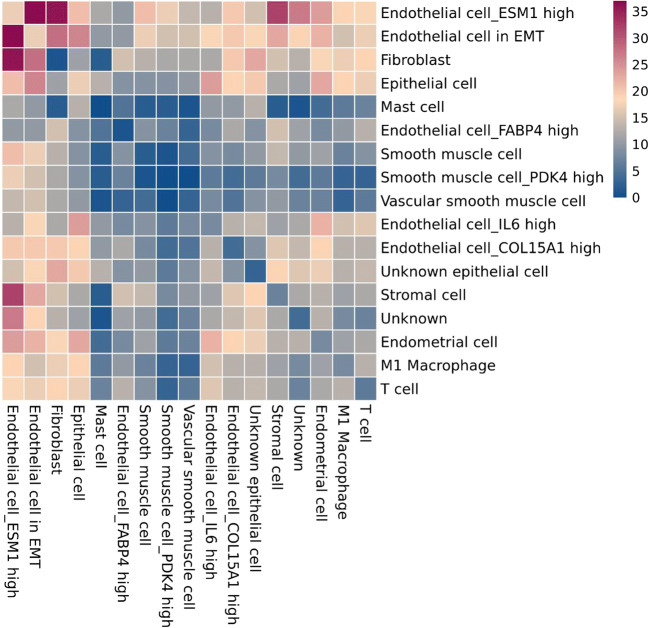
Heatmap of uterine cell-cell interaction

Since IUA is mainly the result of fibrotic lesions of endometrial injury, we assessed the interactions between various types of cells in the uterus. Direct interaction between endometrial cells and most other types of cells was weak (Fig. 6), which implies that when the endometrial cells are injured, it is difficult for other cells to receive relevant signals and dedifferentiate or transdifferentiate into endometrial cells, supporting our proposed insufficient cell plasticity model described in “Insufficient Cell Plasticity Model for IUA Pathogenesis.” Likewise, immune cells (T cell, M1 macrophage, and mast cell) in the uterus interacted with other cells weakly. It is not known whether this is beneficial or not because cells like macrophages have been reported for their dual roles in disease progression and protection [81, 82].

In contrast, the interaction between endothelial cells with others was high. In particular, fibroblasts, stromal cells, and epithelial cells were major targets. It can be conjectured that injured endometrial cells may communicate with other cells via endothelial system and fibroblasts would be the very first one to respond, due to strong interaction. It is worth noting that the endothelial cell includes “endothelial cell in EMT” (Fig. 6), which transforms into mesenchymal cells under normal physiological conditions. After the endometrium is injured, EMT can lead to the disintegration of microvascular structures and closely packed epithelioid tissues of the endometrium. Similar pathological development was observed in atherosclerotic lesions [83].

Taking both cell plasticity and cell-cell interaction into consideration, we hypothesized a transdifferentiation route of pericytes and endothelial cells to fibroblasts during the formation of IUA, which is shown in Fig. 8.

## Stem Cell–Related Therapies for IUA and Its Molecular Mechanisms

### Research Progress and Limitations

Many studies have confirmed that transplantation of bone marrow–derived stem cells, hematopoietic stem/progenitor cells, endometrial MSCs, embryonic stem cells, amniotic MSCs, and blood-derived stem cells can effectively promote the endometrial regeneration and menstrual recovery of IUA animal models or patients [84–86]. To improve the endometrial receptivity and improve the therapeutic effect of stem cell transplantation, Zhang et al. [87] combined platelet-rich plasma (PRP) and blood-derived stromal cells in the IUA rat model in order to explore the therapeutic mechanisms. They suggested that menstrual blood–derived stromal cells had a significant effect on the Hippo pathway of endometrial cells and then significantly affected the expression of downstream molecules CTGF, Wnt5a, and Gdf5.

Research on stem cell therapy treating IUA was limited to proving that stem cells are beneficial to the repair of endometrium. Except for the abovementioned study by Zhang et al., there were few studies of stem cell therapy studies for IUA focusing on the molecular mechanism. However, the understanding of molecular mechanism is urgently needed to improve the efficacy of stem cell treatment.

### Stem Cell–Derived Extracellular Vesicle Therapy

#### Feasibility and Advantages

Extracellular vesicles are a heterogeneous group of membrane-structured particles derived from cells, including exosomes and microvesicles. They originate from the endosomal system or shedding from the plasma membrane, respectively. They contain proteins, mRNA, and microRNA and involve in intercellular substance exchange and signal transmission. Stem cells were found to affect the repairing of injured cells mainly through paracrine actions [88–91], in which extracellular vesicles (EVs) play a key part. EVs released by MSCs during tissue repair contain paracrine factors such as chemokines, growth factors, and cytokines, which have anti-inflammatory, anti-scarring, and pro-angiogenic effects [92].

Stem cell therapy for IUA is still in the clinical trial stage with very long treatment cycle. If obtaining the patients’ stem cells by bone marrow aspiration, the patients have to undergo at least two invasive treatments of bone marrow aspiration plus hysterectomy. Ethical and psychological issues pose a challenge for patients [93]. The uncertainty and experimentation of stem cell therapy raise many ethical concerns. With the extensive development of stem cell–related clinical trials, the International Stem Cell Society has even published related books for patients seeking stem cell therapy to popularize the psychological preparation and purpose of stem cell therapy [94]. How to apply the easy-to-accept IUA treatment method to the clinic is an urgent problem of assisted reproductive medicine. If stem cell–derived EVs can be isolated from culture solution and then injected intravenously, it will greatly facilitate the treatment.

#### Research Progress and Limitations

There was only one case study of stem cell–derived EV therapy for IUA, in which enriched EVs from human umbilical cord mesenchymal stem cell culture medium were injected into the right uterine horn of IUA model rats [95]. The results showed that both inflammation and fibrosis in rats were significantly reduced, and angiogenesis was also significantly improved. However, no molecular mechanisms were studied.

### Stem Cell–Related Therapies for IUA and Cell Plasticity

**Fig. 7:**
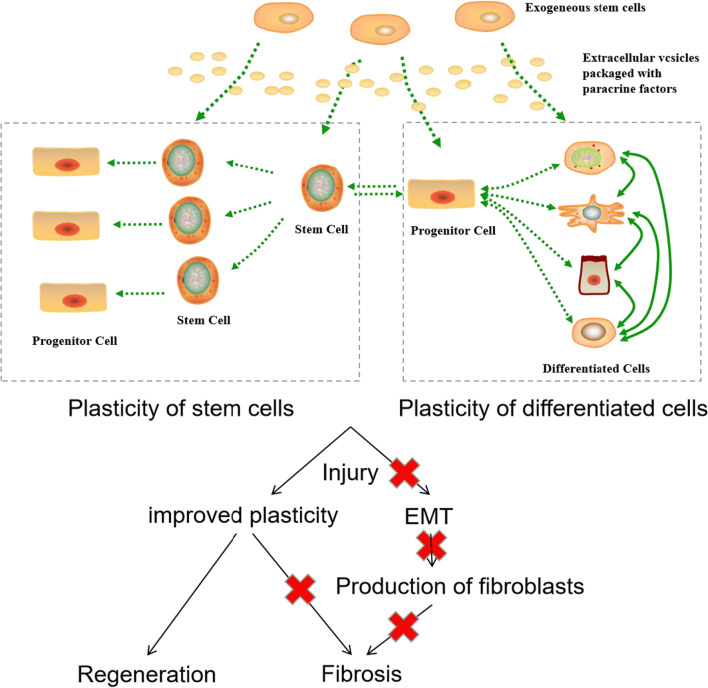
Hypothesized repair mechanism by stem cell therapy

**Fig. 8:**
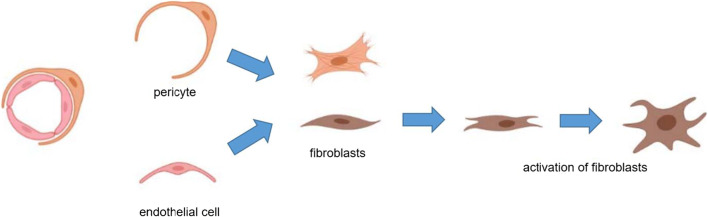
Hypothesized transdifferentiation of pericytes and endothelial cells to fibroblasts

Mesenchymal stem cell–based therapy mainly repairs injured tissues through paracrine signaling (described in “Research Progress and Limitations”). Stem cells also secrete EVs, which are packaged with functional molecules and effect on target cells [90, 95]. Therefore, the molecular pathways mediated by stem cells or stem cell–derived EVs for damaged tissue repair should be similar. Self-repair of injured tissue depends on the plasticity of differentiated and stem cells (described in “Uterine Cell Heterogeneity”). A possible scenario is that EVs of MSCs improve the plasticity of cells in the injured endometrium, thereby promoting tissue regeneration (Fig. 7). The influence of EVs on the plasticity of target cells is thus a promising primer towards elucidating the molecular mechanisms of stem cell–related therapy. By first questioning the key components in stem cell–derived EVs, followed by identifying the molecules and signaling pathways associated with cell plasticity, mechanistic studies could then be designed to interrogate the key players involved in the repair of the injured endometrium (Fig. 8).

There were studies on searching for paracrine factors functioning in the repair of various injured tissues in animal models, though no related research on the endometrium; they still provide candidates for further studies. For example, in the study of rat models, TGF-β, FGF-2, angiopoietin-2, IGF-1, VEGF, EGF, bFGF, SDF-1, HGF, and IL-6 were considered to be the main paracrine factors [84, 96–98]. Likewise, in the study of mouse models, NGF, HGF, IL-10, TGF-β1, VEGF, IGF-1, angiogenin, and IL-8 were considered to be the main paracrine factors [99–101]. Common factors between these studies include VEGF, TGF-β, and IGF.

Many studies on the molecular mechanisms of repairing by EVs mainly focused on the skin. Molecules in EVs could affect different molecules/signaling pathways in different types of cells in the skin. MiRNA-21, miRNA-23a, miRNA-125b, and miRNA-145 in EVs activated AKT, ERK1/2, Wnt4/β-catenin, and STAT3 and inhibited TGF-β/SMAD2 in fibroblasts [102–107]. MiRNA-181c and Let-7b in EVs inhibited TLR4 and NF-κB and activated STAT3 and AKT in macrophages [108–110]. EVs activated AKT and Wnt4/β-catenin in keratinocytes [104, 105, 111, 112]. MiRNA-126-3p in EVs activated AKT, ERK1/2, and Wnt4/β-catenin in endothelial cells [113–115]. Once again, these molecules or pathways can be good starting points for molecular mechanistic studies on EV-treated injured endometrium. Research on the molecular mechanisms of injury-induced plasticity and inflammation-induced plasticity (mentioned in “Insufficient Cell Plasticity Model for IUA Pathogenesis”) should provide further insights.

## Conclusions and Discussion

Routine prevention strategies and treatments for IUA were unsatisfactory and reported efficacy from different studies varies. Correlation studies by differentially expressed genes between endometrial tissue of IUA patients/animal models and normal endometrial tissue have not provided conclusive results to innovate effective therapeutics. Stem cell–related therapies, though appeared promising, yet again suffer from largely animal studies and ambiguities from multicellular structures of the endometrium. To overcome the bottleneck, cell plasticity should receive greater attention, and cell-based models or experimental methods will help dissect the problem and understand the molecular mechanisms in both IUA pathogenesis and treatment.

Specifically, comparing the single-cell expression profile of IUA endometrium and normal endometrium should be powerful in providing new molecular information on IUA pathogenesis. Sing-cell methods can also be used to identify molecular markers of subpopulations in endometrium. Those markers are essential tools in lineage tracing to investigate the dedifferentiation and transdifferentiation of different subpopulations of endometrial cells. If the plasticity of endometrial cells could be proved to be insufficient compared to other rapidly regenerating tissues (e.g., liver, skin), the mechanism of IUA pathogenesis in cell level could be revealed.

## Material and Methods

We conducted literature review in January 2020 by searching articles at PubMed on prevention and treatment, pathogenesis, the repair of other tissues/organs, cell plasticity, and the stem cell–related therapies for intrauterine adhesion. A total of 110 articles were selected for review.

Analysis of uterine cell heterogeneity was conducted via the Human Cell Landscape (HCL) platform (http://bis.zju.edu.cn/HCL/index.html). The atlas of different types of cells in the uterus was based on the t-SNE method.

We reviewed the studies on IUA pathogenesis and selected genes that are considered to be related to IUA pathogenesis in at least two studies, or only in one study in which both the results of IUA patients and IUA animal model show that the genes related to IUA pathogenesis. These genes are combined to form a “gene combination related to IUA pathogenesis.” Based on the uterine single-cell sequencing data, the expression profiles of these genes in each cell type were obtained.

Uterine single-cell sequencing of Chinese population data were retrieved from GEO database (GEO accession number GSE134355). The table listing the barcode sequence and corresponding cell type was obtained from https://figshare.com/articles/HCL_DGE_Data/7235471. Interaction between different cell types was done at https://www.cellphonedb.org/explore-sc-rna-seq.

## Data Availability

The data sets used and analyzed during the current study are stated in the section of “Material and Methods” as shown above.
